# Vaccination with Recombinant Adenoviruses Expressing the Peste des Petits Ruminants Virus F or H Proteins Overcomes Viral Immunosuppression and Induces Protective Immunity against PPRV Challenge in Sheep

**DOI:** 10.1371/journal.pone.0101226

**Published:** 2014-07-11

**Authors:** José M. Rojas, Héctor Moreno, Félix Valcárcel, Lourdes Peña, Noemí Sevilla, Verónica Martín

**Affiliations:** Centro de Investigación en Sanidad Animal (CISA-INIA), Instituto Nacional de Investigación y Tecnología Agraria y Alimentaria, Valdeolmos, Madrid, Spain; Federal University of São Paulo, Brazil

## Abstract

Peste des petits ruminants (PPR) is a highly contagious disease of small ruminants caused by the Morbillivirus peste des petits ruminants virus (PPRV). Two recombinant replication-defective human adenoviruses serotype 5 (Ad5) expressing either the highly immunogenic fusion protein (F) or hemagglutinin protein (H) from PPRV were used to vaccinate sheep by intramuscular inoculation. Both recombinant adenovirus vaccines elicited PPRV-specific B- and T-cell responses. Thus, neutralizing antibodies were detected in sera from immunized sheep. In addition, we detected a significant antigen specific T-cell response in vaccinated sheep against two different PPRV strains, indicating that the vaccine induced heterologous T cell responses. Importantly, no clinical signs and undetectable virus shedding were observed after virulent PPRV challenge in vaccinated sheep. These vaccines also overcame the T cell immunosuppression induced by PPRV in control animals. The results indicate that these adenovirus constructs could be a promising alternative to current vaccine strategies for the development of PPRV DIVA vaccines.

## Introduction

Peste des petits ruminants (PPR) is a very important and contagious disease of small ruminants, mainly sheep and goats, notifiable to the World Organization for the Animal Health (OIE). The disease is endemic in Asia, the Middle East and Africa, and is spreading to other countries, as evidenced by the recent outbreak declared in December 2013 in China. It causes significant economical losses in endemic regions. Clinically PPR may vary from acute infection with severe clinical disease and death to mild, with little or no visible clinical signs. Acute infection may include severe pyrexia [41.0–41.3°C] with affected animals often becoming restless, having a dull coat, dry muzzle, catarrhal inflammation of the ocular and nasal mucosa, diarrhoea, enteritis, pneumonia and loss of appetite [1]. The mortality rate is comprised between 50–80% in the acute cases [2]. The eradication in 2001 of the closely related Rinderpest Virus (RPV) has increased the global interest in PPRV because of its emergence and has highlighted the necessity to develop specific strategies for its surveillance and prevention through vaccination [3]. The causal agent, Peste des petits ruminants virus (PPRV), belongs to the genus *Morbillivirus*, in the family Paramyxoviridae. There is only a single serotype of PPRV but it is genetically grouped into four distinct lineages (1, 2, 3 and 4) on the basis of partial sequence analysis of fusion protein (F) gene [4], [5], [6]. PPRV is an enveloped negative single strand RNA virus with two external glycoproteins, F and hemagglutinin (H), associated with the envelope [7]. These represent key antigens for triggering an effective protective immune response. PPRV is a lymphotropic virus, causing leucopenia and a generalized immunosuppression [3]. Current PPRV vaccines [8] are based on live virus attenuated by serial passage in Vero cells of various PPRV strains (Nigeria 75/1 [9], [10], Sungri'96, Arasur'87 and Coimbatore'97 [11]) and they are extensively used in countries where PPRV is endemic [12]. Single immunization with live PPRV vaccines has been able to maintain protective levels of serum antibody for up to three years. Although effective, an important drawback of this vaccine is that vaccinated animals cannot be differentiated from infected animals, affecting control and regulatory measures against the disease. They are also thermo-sensitive and require an efficacious cold chain to remain active, which is an important practical problem, more so in developing countries and warm climates, where the disease is more prevalent. Different research groups are focused on developing thermo-resistant live attenuated vaccines to overcome the obstacles posed by having to maintain the cold chain for vaccine distribution [13]. Several strategies that allow the expression of the F and/or H proteins of PPRV using different vectors, including recombinant adenoviral [14], [15] or poxviral vectors [16], [17], and chimeric RPV [18], [19] have been shown to induce long lasting neutralizing antibody responses against PPRV in goats as well as partial or total protection against disease in some cases. Adenoviruses have proved excellent candidates as vaccine delivery vehicles [20], [21] due to their genetic stability, safety [14] and the strong immune response they generate [9], [10], [22], [23], [24]. Furthermore, they can be easily produced in large quantities and their structural characteristics make them amenable to distribution in hot climates areas, like Africa and Asia, where PPRV is currently a major threat.

Previously, we have generated two recombinant adenoviruses expressing the F or H proteins from PPRV and demonstrated that they induce specific PPRV neutralizing antibodies as well as cellular immune responses to PPRV in mice [25]. In the present study, protective immune response to these two recombinant adenoviruses was evaluated in sheep. PPRV-specific B- and T- cell responses were induced by both recombinant vaccines and protected sheep against virulent challenge. These vaccines overcame the T cell immunosuppression observed in infected animals. These adenovirus constructs are a promising alternative to current vaccine strategies for the development of PPRV DIVA vaccines.

## Material and Methods

### Cells and viruses

Vero-dogSLAM cells (VDS) were obtained from Dr. Parida IAH, Pirbright. PPRV strains Nigeria 75/1 or Ivory Coast'89 (ICV) (lineages I and II), respectively, were obtained from Dr. Batten, IAH Pirbright, and recombinant adenoviruses (Ad5-PPRV-F and Ad5-PPRV-H) were used as detailed in [25].

### Animal Experiments

Two month old-female sheep from the “*Colmenareña*” breed from a certified provider were randomly divided into 4 groups, with 4 sheep per group, and housed in separate rooms with controlled temperature and light/dark cycles. Food and water were provided *ad libitum*. All experiments were carried out in a disease-secure isolation facility (BSL3) at the *Centro de Investigación en Salud Animal* (CISA), in strict accordance with the recommendations in the guidelines of the Code for Methods and Welfare Considerations in Behavioural Research with Animals (Directive 86/609EC; RD1201/2005) and all efforts were made to minimize suffering. Experiments were approved by the Committee on the Ethics of Animal Experiments (CEEA) of the Spanish *Instituto Nacional de Investigación y Tecnología Agraría y Alimentaria* (INIA) and the “*Comisión de ética estatal de bienestar animal*”. An acclimatization period of two weeks was observed, during which animals were daily monitored for general health staus prior to the beginning of the experiment. Animals were inoculated intramuscularly (im) with PBS (group 1; n = 4); with 10^8^ infectious units (IU) of Ad5 (group 2; n = 4); with 10^8^ IU of Ad5-PPRV-F (group 3; n = 4); or with 10^8^ IU of Ad5-PPRV-H (group 4; n = 4). One booster inoculation was performed with the same amount of virus after 21 days. Challenge was performed at day 42 post-immunization by intravenous inoculation of 10^6^ plaque forming units (pfu) of ICV'89 PPRV strain. Animals were bled at days 0 (naïve), 7, 21, 28, 42 (pre-challenge) and 3, 5, 7, 11, 13 (post-challenge), and sacrificed at 13 days post-challenge (pc). Euthanasia was performed by intravenous administration of T61 (4–6 ml/50 kg bw) following intramuscular xylazine (0.3 mg/kg bw) inoculation to minimize suffering of sheep. Ocular, nasal and oral swabs were collected at different days pre and post-challenge for detection of PPRV. Animals were daily examined for clinical signs of infection and their rectal temperatures were recorded to ensure that any animal found to be suffering could have been given appropriate veterinary care in accordance with standard veterinary practice. Scores from 0 to 4 for each animal were calculated based on the severity of ocular, oral and nasal congestion and discharge as well as signs of apathy, anorexia, diarrhoea and loss of appetite, using a slightly modified version of the scoring method provided by [26], [27]. Briefly, scores were assigned for each of the following categories: general clinical signs, pyrexic response, ocular/nasal discharge, faeces and respiratory symptoms. The final score obtained for each animal and day was the sum of these, ranging from 0 for a healthy animal to a possible maximum of 20. No animals died during the course of the experiment, neither did they reach severity scores granting euthanasia. A scheme of the experimental design is presented in Table 1.

**Table 1:** Experimental design showing the different animal groups.

Treatment	Group	Sheep #	Vaccine	Route of vaccination	Dose of vaccine per inoculums (IU)	Virulent virus used for challenge	Route of challenge	Dose of challenge (pfu)
PBS	1 (n = 4)	1–4	PBS	Intramuscular	-	Ivory Coast ‘89	Intravenous	10^6^
Ad5	2 (n = 4)	5–8	Ad5	Intramuscular	10^8^	Ivory Coast ‘89	Intravenous	10^6^
Ad5-PPRV-F	3 (n = 4)	9–12	Ad5-PPRV-F	Intramuscular	10^8^	Ivory Coast ‘89	Intravenous	10^6^
Ad5-PPRV-H	4 (n = 4)	13–16	Ad5-PPRV-H	Intramuscular	10^8^	Ivory Coast ‘89	Intravenous	10^6^

### Serum and peripheral blood mononuclear cells (PBMCs) preparation and storage

Blood samples were taken directly from the jugular vein into Venojet glass tubes, without anticoagulant on days 0 (naïve), 7, 21, 28, 42 (pre-challenge), 3, 5, 7, 11 and 13 (post-challenge) and allowed to clot overnight at 4°C. Serum was obtained by centrifugation at 3000 rpm for 10 minutes (min) at 4°C, aliquoted and stored at −80°C until use. PBMCs were obtained from total blood collected in EDTA and purified by Ficoll cushion (GE Healthcare) purification as described previously [28].

### RNA extraction and quantitative PCR (qPCR)

Total RNA extraction from blood samples using Trizol (Invitrogen) as well as reverse transcriptions of the N-mRNAs fragments with SuperScript III reverse transcriptase (Invitrogen) were performed according to the manufacturer's protocols. Quantifications were performed on a LightCycler 96 thermocycler (Roche Applied Science) using the Light Cycler FastStart DNA Master SYBR green I kit (Roche Applied Science). The total N RNA fragment was used as standard. This was obtained as a runoff transcript from a molecular DNA clone encoding the N in the genomic sense, cloned into pGem-T Easy Vector (Promega) to provide the corresponding standard curves in the qPCR reaction. The N-ICV'89 region was amplified with primers Forward-5′ AGAGTTCAATATGTTATTAGCATCCAT-3′ and Reverse-5′ TTCCCCAATCACTCTCCTCTGT-3′. Each value of the amount of PPRV RNA is the average of at least three independent determinations.

### Anti-PPRV IgG ELISA

Anti-PPRV IgG ELISA were adapted from a method previously described [25]. Briefly, ELISA plates (Maxisorp, Nunc) were coated with purified PPRV Nigeria 75/1 overnight at 4°C (equivalent to 10^4^ pfu per well). After blocking with 10% FCS in PBS and washing three times with 0.1% Tween in PBS, sera from inoculated sheep diluted in PBS +1% FCS were applied to the plate. The presence of PPRV-specific IgGs was detected using a secondary Donkey anti-sheep IgG conjugated with horseradish peroxidase (Serotec) diluted 1∶6666 in PBS +0.5% FCS. After washing ten times with 0.1% Tween in PBS, signal was developed using 3,3′,5,5′-Tetramethylbenzidine (TMB) Liquid Substrate System (Sigma) and the reaction was stopped with 3 M sulphuric acid before reading. Optical density (OD) was determined at 450 nm on a FLUOstar Omega (BMG Labtech) ELISA plate reader. All IgG measurements were made in triplicate and assays were only considered valid when standard deviations were below 10% of the average. IgG binding to PPRV was considered positive only when OD in the test well was at least twice the OD obtained with the pre-immune serum from the same sheep. Thus, anti-PPRV IgG titre in serum was defined as the serum dilution necessary to achieve readings twice that of pre-immune serum and was calculated using a linear regression of serum dilutions vs. OD readings at 450 nm. Data are presented as the average (±SEM) IgG titre for each treatment group.

### Virus Neutralization Test (VNT)

Serum samples were inactivated for 30 min at 56°C and tested for the presence of neutralizing antibodies as described previously [29], [30]. Briefly, Nigeria 75/1 PPRV stock was incubated with serial dilutions of inactivated sheep serum for 1 hour (h) at RT in triplicate. The mixtures were added to VDS cells, incubated for 5 days, fixed with 2% formaldehyde and cells visualized by crystal violet staining.

### PPRV -Competition and capture ELISA

Sera obtained from sheep at different times were analysed by a commercial competition ELISA [IdVet: ID Screen PPR Competition (PPRC)] for the detection of antibodies against the nucleoprotein (N) of PPRV [31]. The presence of PPRV in swabs obtained at different times from sheep were analysed by a commercial capture ELISA [IdVet: ID Screen PPR Antigen Capture (PPRAG)]. The experiments were performed following the manufacturer's instructions.

### IFNγ - ELISPOT assays

Ovine IFN-γ ELISPOT assays were performed using MSIPS4510 plates (Millipore). Membranes were activated using sterile 35% ethanol for 1 min, and after thorough washing with sterile water, incubated overnight at 4°C with 5 µg/ml anti-ovine IFN-γ antibody (MT17.1, Mabtech, Sweden). Plates were blocked in RPMI (supplemented with 17% AIM-V [Gibco/life technologies], glutamine, Na^+^-pyruvate, HEPES, non-essential amino acids, antibiotics and 10% FCS) for 2 h at room temperature. Sheep PBMCs were then plated at a density of 2–3 x 10^5^ cells per well and incubated with BEI-inactivated PPRV (Nigeria 75/1 and ICV'89 strains), PBMC medium as negative control or Concanavalin-A (Con-A) (0.5 µg/ml) as positive control for 48 h at 37°C, 5% CO_2_. After discarding the cells and washing with PBS, membranes were incubated with biotin-labelled anti-ovine IFN-γ antibody (MT307-biotin, Mabtech, Sweden) diluted at 0.25 µg/ml in PBS +0.5% FCS for 2 h. After 5 washes in PBS, membranes were incubated for 1 h with streptavidin conjugated to alkaline phosphatase (ExtrAvidin-AP, Sigma) diluted 1∶10,000 in PBS+0.5% FCS. Membranes were washed thoroughly first in PBS and then in distilled water before ELISPOT assay reactions were developed using Sigma FAST BCIP/NBT (Sigma). Once spots were formed, membranes were washed with abundant distilled water and allowed to air dry in the dark. All cultures were performed in triplicates and ELISPOT assays were considered valid only when average spot counts were below 25 for control cultures and standard deviations in positive wells below 15% of the average counts.

### Lymphocyte Proliferation assays

Sheep PBMCs were plated at a density of 3x10^5^ per well in flat bottom 96-well plates in the presence of BEI-inactivated PPRV (equivalent to 1 x 10^4^ pfu per well prior to inactivation) and incubated at 37°C, 5% CO_2_ at a 5^0^ angle. In all experiments, cells were cultured with medium or VDS cell lysate as negative control and with Con-A (0.5 µg/ml) as positive control. All cultures were performed in triplicates. ^3^H -thymidine was added to each well at 5 µCi/ml final concentration on day 5 and incubated overnight. Cells were harvested onto UniFilter 96 filtermat (Perkin-Elmer) using a cell harvester. After drying, scintillation liquid was added and the filtermat were counted using a 1450 MicroBeta Trilux counter (Perkin-Elmer). Data are presented as stimulation index defined as the ratio of incorporated ^3^H-thymidine in test to control cultures.

### Haematology

Haematological parameters -total white cell blood count (WBC), total red cell blood count (RBC), hemoglobin (HGB), hematocrit (HCT), mean cell volume (MCV), mean cell hemoglobin (MCH), mean cell hemoglobin concentration (MCHC), red cell distribution width (RDW), platelet count (PLT), mean platelet volume (MPV), platelet distribution width (PDW) and platelet crit (PCT)- were calculated in theses samples on an Auto Hematology Analyzer (Mindray Bc-2800Vet) running veterinary software.

### PBMC population analysis by flow cytometry

Purified PBMCs were washed in flow cytometry buffer [PBS+2% FCS+0.02% sodium azide (Sigma)] twice and incubated on ice with antibodies for 20 min. Anti-sheep CD4-FITC, CD8-PE, CD14-Alexa647 and IgM-FITC antibodies (all from Serotec) were used in these experiments to detect the CD4^+^ T cells, CD8^+^ T cells and B cells populations in PBMCs, respectively. After the incubation, cells were washed twice in flow cytometry buffer and acquisition was done on a FACScCalibur cytometer (Becton Dickinson). Appropriate isotype controls were used to exclude non-specific binding isotype binding to ovine PBMC and fluorescence minus one approach was used as gating strategy. Data were analysed using FlowJo software (Tree Star Inc.).

### Statistical analyses

Unpaired two-tailed Student's *t*-tests were used to evaluate differences within the same animal, whereas non-parametric two-tailed Mann Whitney rank *U* tests or two-way ANOVA were used to compare treatment groups. Responses to Con-A and IgG titres at different time points were compared using a Wilcoxon matched-pairs signed-rank test. Data handling analyses was performed using Prism 6.0 (GraphPad Software Inc. San Diego, CA, USA).

## Results

### Vaccination with recombinant Ad5 virus expressing the F or H protein induces PPRV specific IgG in sheep

**Figure 1:**
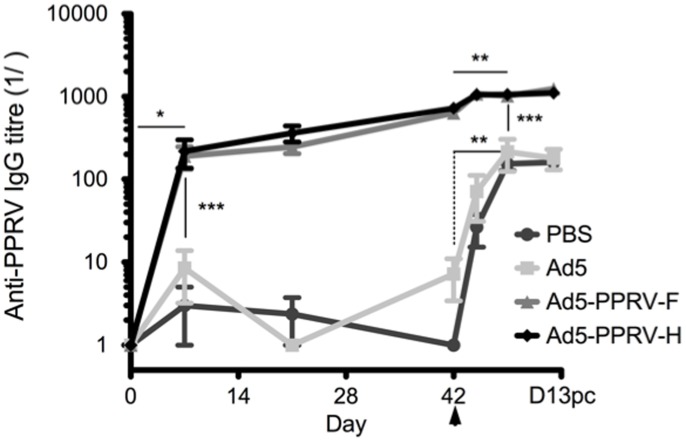
Inoculations with Ad5-PPRV-F or Ad5-PPRV-H induce the production of PPRV-specific IgG in sheep. Groups (n = 4) of sheep were inoculated intramuscularly with PBS, control adenovirus (Ad5), Ad-PPRV-F or Ad-PPRV-H at days 1 and 22 and all animals were challenged with virulent PPRV IVC 89 at day 42 (indicated by arrow). Serum samples obtained at the indicated time points were analysed for PPRV-specific IgG by ELISA using Nigeria 75/1 PPRV coated plates. Data are presented as average (+/−SEM) IgG titre for each treatment group. * p<0.01 in Wilcoxon matched-pairs signed rank test; day 7 vs day 0 in immunized sheep.** p<0.01 in Wilcoxon matched-pairs signed rank test; day 7 post-challenge vs day 42 pre-challenge. *** p<0.001 Mann-Whitney test: vaccinated (Ad5-PPRV-F and Ad5-PPRV-H) vs control (PBS and Ad5) sheep at the same time point.

To determine whether the recombinant adenoviruses expressing PPRV F or H proteins elicited a specific immune response in sheep, groups of four animals were inoculated im with PBS, control adenovirus (Ad5) or Ad-PPRV-F or Ad-PPRV-H (Table 1). A booster inoculation was performed 21 days later. Sera were tested for the presence of PPRV-specific IgG by ELISA (Figure 1). Sheep inoculated with Ad5-PPRV-F or Ad5-PPRV-H developed PPRV-specific IgG as early as day 7 post-vaccination, whereas no virus-specific IgG were detectable in control sheep (PBS and Ad5). By the time of challenge on day 42, PPRV-specific IgG titres in vaccinated sheep increased by at least 2 logarithmic units (Figure 1).

At day 42 post-vaccination, sheep were challenged with the virulent ICV'89 PPRV strain and further serum samples were collected at days 3, 7 and 13 post-challenge (pc). PPRV challenge on day 42 resulted in rapid and significant production of virus-specific IgG in control animals and a slight increase in IgG titers was also detected in vaccinated animals. Importantly, PPRV-specific IgG levels in challenged control sheep remained at least one logarithmic unit below their vaccinated counterpart. These data show that recombinant Ad5 virus expressing PPRV proteins F or H can induce high magnitude virus-specific IgG response in vaccinated sheep.

### Induction of specific neutralizing antibody response against PPRV in sheep inoculated with Ad-5-PPRV-F and Ad-5-PPRV-H

To determine the presence of neutralizing antibodies, sera from vaccinated sheep described above were assayed in a virus neutralization test using PPRV Nigeria 75/1. As shown on Table 2, three out of four animals vaccinated with a single inoculation of Ad5-PPRV-H and all sheep vaccinated with a single dose of Ad5-PPRV-F showed PPRV-specific neutralizing antibodies titers at day 21 post-vaccination. After the booster inoculation, all vaccinated sheep (8) showed PPRV-specific neutralizing antibodies. As expected, no neutralization activity was detected in preimmune sera or sera from sheep inoculated with either PBS or Ad5 prior to challenge. After challenge with PPRV, the neutralization titers increased in all infected groups. Vaccinated animals (groups 3 and 4) showed values ranging from 4–64 as early as day 3 pc, which rapidly increased to values ranging from 200–800 by day 13 pc. In contrast, control sheep (goups 1 and 2) only showed neutralization antibody activity from day 5 pc with values ranging from 4–16. Their neutralization titers increased more slowly and reached lower values by day 13 pc (100–400), than those obtained from vaccinated sheep sera. Thus, a single inoculation of recombinant adenoviruses expressing either the PPRV F or H proteins is able to induce PPRV neutralizing antibodies, with a second booster inoculation significantly increasing the neutralization titers.

**Table 2:** Analysis of PPRV neutralization activity in sera from vaccinated and non vaccinated sheep.

Neutralization titer^a^									
Treatment^b^	Sheep #	D0^c^	D21^d^	D42^d^	D3PCh^e^	D5PCh^e^	D7PCh^e^	D11PCh^e^	D13PCh^e^
PBS	1	<2	<2	<2	<2	16	40	160	200
	2	<2	<2	<2	<2	16	80	80	200
	3	<2	<2	<2	<2	16	80	160	200
	4	<2	<2	<2	<2	16	80	320	400
Ad5	5	<2	<2	<2	<2	16	80	160	200
	6	<2	<2	<2	<2	4	80	80	400
	7	<2	<2	<2	<2	16	160	160	400
	8	<2	<2	<2	<2	16	20	80	100
Ad5-PPRV-F	9	<2	4	4	8	64	160	320	400
	10	<2	4	8	8	32	160	160	200
	11	<2	4	8	8	64	160	320	400
	12	<2	4	4	64	64	160	320	400
Ad5-PPRV-H	13	<2	4	4	16	64	160	320	400
	14	<2	2	4	4	8	20	80	800
	15	<2	<2	16	16	64	160	320	800
	16	<2	2	4	4	64	160	320	800

a SN titers were determined against Nigeria 75/1 PPRV strain and expressed as the reciprocal of the last dilution of serum that neutralized 50% of the virus-specific cytopathic effect in flat bottom 96 well plates.

b Intramuscular inoculations of PBS, Ad5 or vaccine vectors as described in the Material and Methods section (See Table 1).

c Sera obtained from uninfected sheep.

d Sera obtained from vaccinated sheep after 21 days post first immunization and 21 days post second immunization (42 days post first immunization).

e Sera obtained from infected sheep after days 3,5,7,11,13 post challenge.

### Vaccination with Ad5-PPRV-F or Ad5-PPRV-H elicits specific PPRV T cell responses

**Figure 2:**
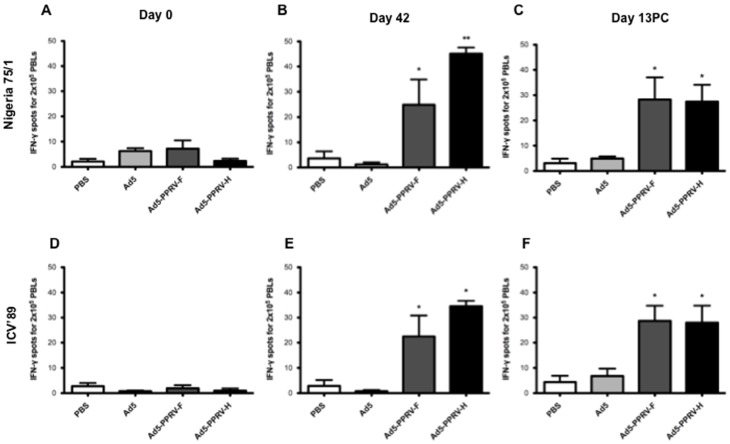
Specific IFN-γ production to F- and H-PPRV proteins in PBMCs from Ad5-PPRV-F to Ad5-PPRV-H inoculated sheep detected by ELISPOT assay. PBMCs from sheep inoculated with PBS, Ad5, Ad5-PPRV-F or Ad5-PPRV-H were isolated at 0 and 42 day post vaccination and 13 days post challenge and cultured for 48 h in the presence of BEI inactivated Nigeria 75/1 (panels A, B, C) or ICV'89 (panel D, E, F) PPRV strains virus. The production of IFN-γ was measured using an ELISPOT assay. Data are presented as average (+/− SEM) IFN-γ spots above background for each treatment group. Assays were considered valid only when IFN-γ spot counts in control wells were below 25 for 2×10^5^ cells, and standard deviations in positive wells below 15% of the average. A positive control of PBMCs activated with 0.5 µg/ml Con-A (Sigma) was always included to validate the ELISPOT assay. * p<0.05 and ** p<0.01 Mann–Whitney test.

**Figure 3:**
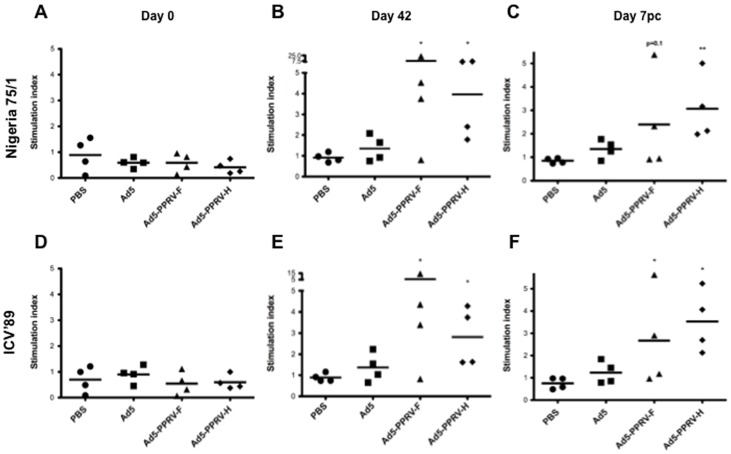
Specific proliferation in PBMCs from Ad5-PPRV-F and Ad5-PPRV-H vaccinated sheep. Sheep PBMCs collected on days 0 (A and D panels), 42 post vaccination (B and E panels) and 7 post-challenge (C and F panels) were stimulated for 6 days either with Nigeria 75/1 (A, B, C panels) or ICV'89 (D, E, F panels) PPRV strains. Cell proliferation detected by [^3^H]-thymidine incorporation is represented. The stimulation index was calculated as the count ratio between PPRV and negative control PBMC cultures. Data are presented as average stimulation index for each sheep in each treatment group. The line indicates the average proliferative response to PPRV in the treatment group. (* p≤0.05, ** p<0.01 Mann-Whitney test Ad5-PPRV-F or Ad5-PPRV-H vs PBS).

In order to establish whether Ad5-PPRV-F and Ad5-PPRV-H vaccination induced specific anti PPRV cellular responses, PBMCs from vaccinated sheep obtained on days 0 (naïve), 42 post-vaccination and 7 or 13 pc were tested for IFN-γ production (Figure 2) and proliferation (Figure 3) to PPRV. As expected, naïve sheep PBMC did not produce IFN-γ nor proliferate in response to PPRV (Figure 2 A and D, Figure 3 A and D). On day 42, groups vaccinated with Ad5-PPRV-F or Ad5-PPRV-H produced IFN-γ and proliferated specifically to the virus, whereas no PPRV-specific IFN-γ or proliferation was detected in control sheep (PBS or Ad5) (Figure 2 B, Figure 3 B). Interestingly, Ad5-PPRV-F and Ad5-PPRV-H elicited T cell responses, not only to PPRV Nigeria 75/1 from which genes the recombinant vaccine was derived, but also to PPRV from a different genetic lineage (PPRV ICV'89) (Figure 2 E, Figure 3 E). Likewise, IFN-γ production and proliferation to both PPRV Nigeria 75/1 and ICV'89 strains were detected in vaccinated sheep after the virus challenge. No responses to PPRV after challenge were detected from non-vaccinated sheep (Figure 2 C and F, Figure 3 C and F). Taken together, these data demonstrate that vaccination with Ad5-PPRV-F or Ad5-PPRV-H elicits PPRV-specific T cell responses against cognate and heterologous virus strains in sheep.

### Ad5-PPRV-F and Ad5-PPRV-H vaccination in sheep allows differentiation from PPRV infected animals

Infection with PPRV or conventional vaccines against PPRV in sheep is known to induce antibodies against the PPRV-N. To confirm that the use of recombinant adenoviruses allows differentiating vaccinated from infected animals, we analysed the sera described above for the presence of antibodies against the PPRV-N protein using a commercial ELISA procedure. No anti-N antibodies were detected in Ad5-PPRV-F or Ad5-PPRV-H vaccinated animals neither in the control groups (1 and 2) before the challenge (Table 3). As expected, challenge with PPRV resulted in the generation of anti-N antibodies from days 5 or 7 pc in all inoculated animals (Table 3).

**Table 3:** ELISA determination of PPRV N antigen seroconversion in vaccinated and non vaccinated sheep.

Treatment^a^	Sheep #	D0^b^	D42^c^	D3PC^d^	D5PC^d^	D7PC^d^	D11PC^d^	D13PC^d^
PBS	1	-	-	-	+	+	+	+
	2	-	-	-	+	+	+	+
	3	-	-	-	+	+	+	+
	4	-	-	-	+	+	+	+
Ad5	5	-	-	-	+	+	+	+
	6	-	-	-	d^e^	+	+	+
	7	-	-	-	+	+	+	+
	8	-	-	-	+	+	+	+
Ad5-PPRV-F	9	-	-	-	+	+	+	+
	10	-	-	-	+	+	+	+
	11	-	-	-	d^e^	+	+	+
	12	-	-	-	d^e^	+	+	+
Ad5-PPRV-H	13	-	-	-	+	+	+	+
	14	-	-	-	-	+	+	+
	15	-	-	-	+	+	+	+
	16	-	-	-	+	+	+	+

a Intramuscular inoculations of PBS, Ad5 or vaccine vectors as described in the Material and Methods section (See Table 1).

Sera obtained from:

b Non-vaccinated sheep at day 0.

c sheep at 21 days post second immunization (42 days post first immunization) or from.

d PPRV challenged animals at the indicated days.

e No clear positive nor negative PPRV detection.

### Ad5-PPRV-F and Ad5-PPRV-H vaccination prevents PPRV shedding from infected sheep

PPRV is transmitted among infected animals mainly through ocular, nasal and oral discharges [3]. Therefore, we were interested in assessing the presence of PPRV in ocular, nasal and oral swabs obtained at different times after challenge in all experimental groups. As expected, all swabs collected before challenge were negative for PPRV (Table 4). More relevantly, all swabs from vaccinated animals obtained after challenge were also negative for PPRV, suggesting that vaccinated animals are unlikely to shed virus to their surroundings. In contrast, six out of eight non-vaccinated sheep tested were positive for PPRV in at least one of the three swabs collected from each animal at day 7 pc. Moreover, PPRV was detected in swabs from non-vaccinated animals as early as day 5 pc and up today 13 pc, showing a prolonged virus shedding period of PPRV in non vaccinated animals These results indicate that vaccination of sheep with either Ad5-PPRV-F or Ad5-PPRV-H effectively prevents PPRV intra host spreading and therefore possibly virus shedding.

**Table 4:** ELISA detection of PPRV in vaccinated and non-vaccinated sheep.

Treatment^a^	Sheep #	D42^b^			D3PC^c^			D5PC^c^			D7PC^c^			D11PC^c^			D13PC^c^		
	Swab	C^d^	N^e^	O^f^	C^d^	N^e^	O^f^	C^d^	N^e^	O^f^	C^d^	N^e^	O^f^	C^d^	N^e^	O^f^	C^d^	N^e^	O^f^
PBS	1	**-**	**-**	**-**	**-**	**-**	**-**	**-**	**+**	**+**	**+**	**+**	**+**	**-**	**-**	**+**	**-**	**-**	**-**
	2	**-**	**-**	**-**	**-**	**-**	**-**	**-**	**-**	**+**	**-**	**+**	**+**	**-**	**-**	**-**	**-**	**-**	**-**
	3	**-**	**-**	**-**	**-**	**-**	**-**	**-**	**-**	**-**	**-**	**+**	**-**	**-**	**-**	**-**	**-**	**-**	**-**
	4	**-**	**-**	**-**	**-**	**-**	**-**	**-**	**-**	**-**	**-**	**-**	**-**	**-**	**-**	**-**	**-**	**-**	**-**
Ad5	5	**-**	**-**	**-**	**-**	**-**	**-**	**-**	**+**	**-**	**+**	**+**	**+**	**+**	**+**	**-**	**+**	**-**	**-**
	6	**-**	**-**	**-**	**-**	**-**	**-**	**-**	**-**	**-**	**-**	**-**	**-**	**-**		**-**	**-**	**-**	**-**
	7	**-**	**-**	**-**	**-**	**-**	**-**	**-**	**+**	**+**	**+**	**+**	**+**	**+**	**-**	**-**	**-**	**-**	**-**
	8	**-**	**-**	**-**	**-**	**-**	**-**	**+**	**-**	**+**	**+**	**+**	**+**	**+**	**-**	**+**	**-**	**-**	**-**
Ad5-PPRV-F	9	**-**	**-**	**-**	**-**	**-**	**-**	**-**	**-**	**-**	**-**	**-**	**-**	**-**	**-**	**-**	**-**	**-**	**-**
	10	**-**	**-**	**-**	**-**	**-**	**-**	**-**	**-**	**-**	**-**	**-**	**-**	**-**	**-**	**-**	**-**	**-**	**-**
	11	**-**	**-**	**-**	**-**	**-**	**-**	**-**	**-**	**-**	**-**	**-**	**-**	**-**	**-**	**-**	**-**	**-**	**-**
	12	**-**	**-**	**-**	**-**	**-**	**-**	**-**	**-**	**-**	**-**	**-**	**-**	**-**	**-**	**-**	**-**	**-**	**-**
Ad5-PPRV-H	13	**-**	**-**	**-**	**-**	**-**	**-**	**-**	**-**	**-**	**-**	**-**	**-**	**-**	**-**	**-**	**-**	**-**	**-**
	14	**-**	**-**	**-**	**-**	**-**	**-**	**-**	**-**	**-**	**-**	**-**	**-**	**-**	**-**	**-**	**-**	**-**	**-**
	15	**-**	**-**	**-**	**-**	**-**	**-**	**-**	**-**	**-**	**-**	**-**	**-**	**-**	**-**	**-**	**-**	**-**	**-**
	16	**-**	**-**	**-**	**-**	**-**	**-**	**-**	**-**	**-**	**-**	**-**	**-**	**-**	**-**	**-**	**-**	**-**	**-**

a Intramuscular inoculations of PBS, Ad5 or vaccine vectors as described in the Material and Methods section (See Table 1).

b Sera obtained from non-infected sheep at 21 days post second immunization (42 days post first immunization) or from.

c PPRV challenged animals at the indicated days post challenge.

d Conjunctive swabs.

e Nasal swabs.

f Oral swabs.

### Vaccination with Ad5-PPRV-F and Ad5-PPRV-H impairs PPRV replication in hosts

**Figure 4:**
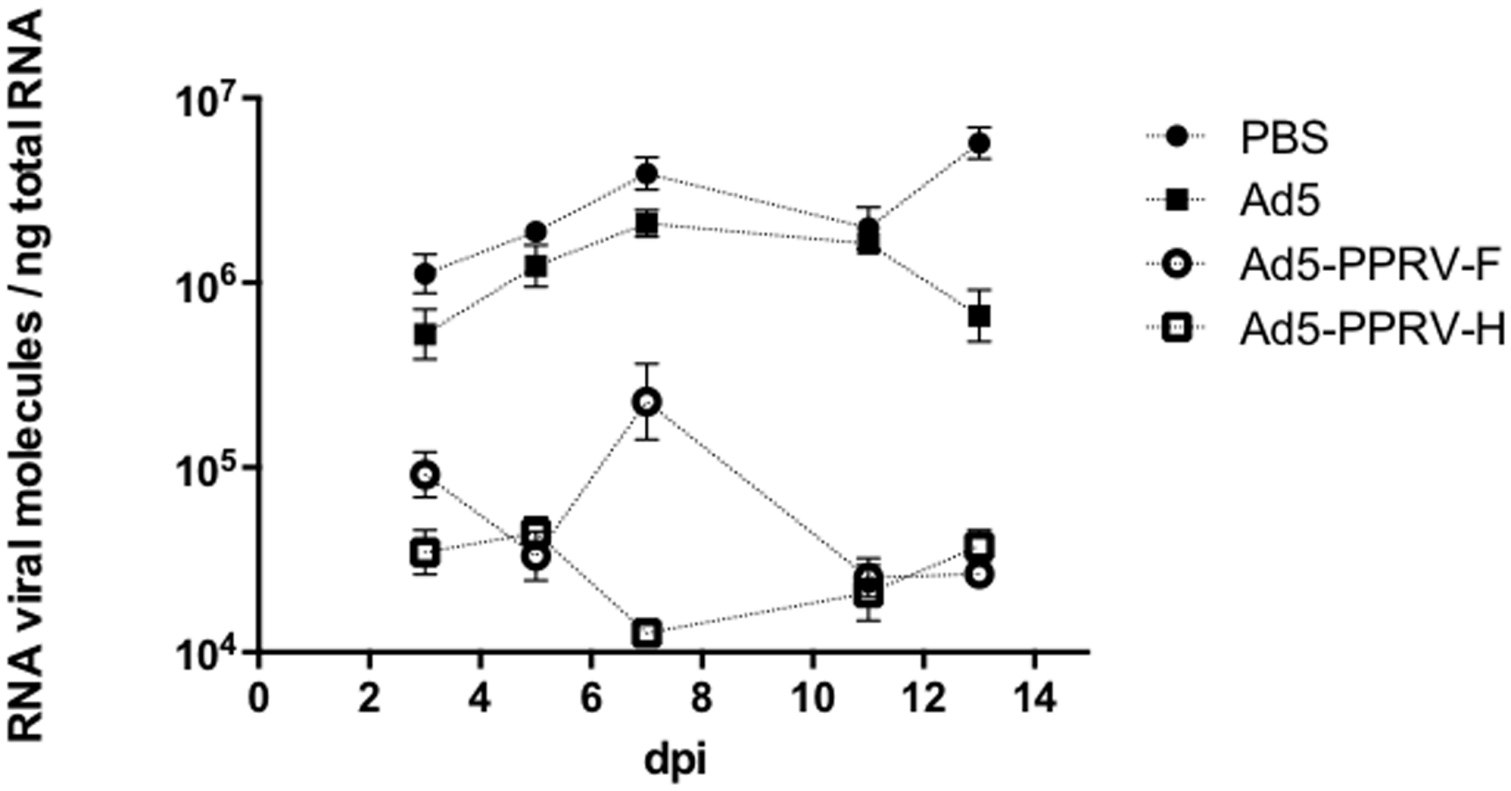
Quantification of viral RNA molecules in blood from vaccinated and non-vaccinated animal groups upon PPRV challenge. The quantification of viral RNA molecules per ng of total blood RNA was performed by qPCR as described in Materials and Methods using triplicates for each sample. Data are presented as average (+/− SEM) for the indicated days post challenge (dpi) for each treatment group as indicated in the legend. Differences were found to be significant (p<0.05; two-way ANOVA) among control (PBS or Ad5-treated) and each vaccinated group at days 5, 7, 11 and 13 post challenge. The qPCR detection limit was 10^4^ RNA viral molecules/ng total RNA.

To determine whether vaccination effectively prevented PPRV viremia in challenged sheep, we used real-time quantitative PCR of the PPRV N gene. Blood samples were obtained at different times from sheep and the number of PPRV messenger RNA molecules in each sample determined. As shown in Figure 4, samples from unvaccinated animals showed detectable levels of PPRV RNA, which peaked at day 7 pc, corresponding with the peak of clinical signs associated to PPRV infection (see below). In comparison, in samples from both groups of vaccinated animals the numbers of PPRV RNA molecules were lower by two logarithmic units than in their unvaccinated counterparts and, importantly, they remained constant throughout the duration of the experiment. This suggests that vaccination of sheep with recombinant Ad5-PPRV-F and Ad5-PPRV-H impairs to a large extent the viremia of PPRV.

### Ad5-PPRV-F or Ad5-PPRV-H vaccination protects against PPRV challenge

**Figure 5:**
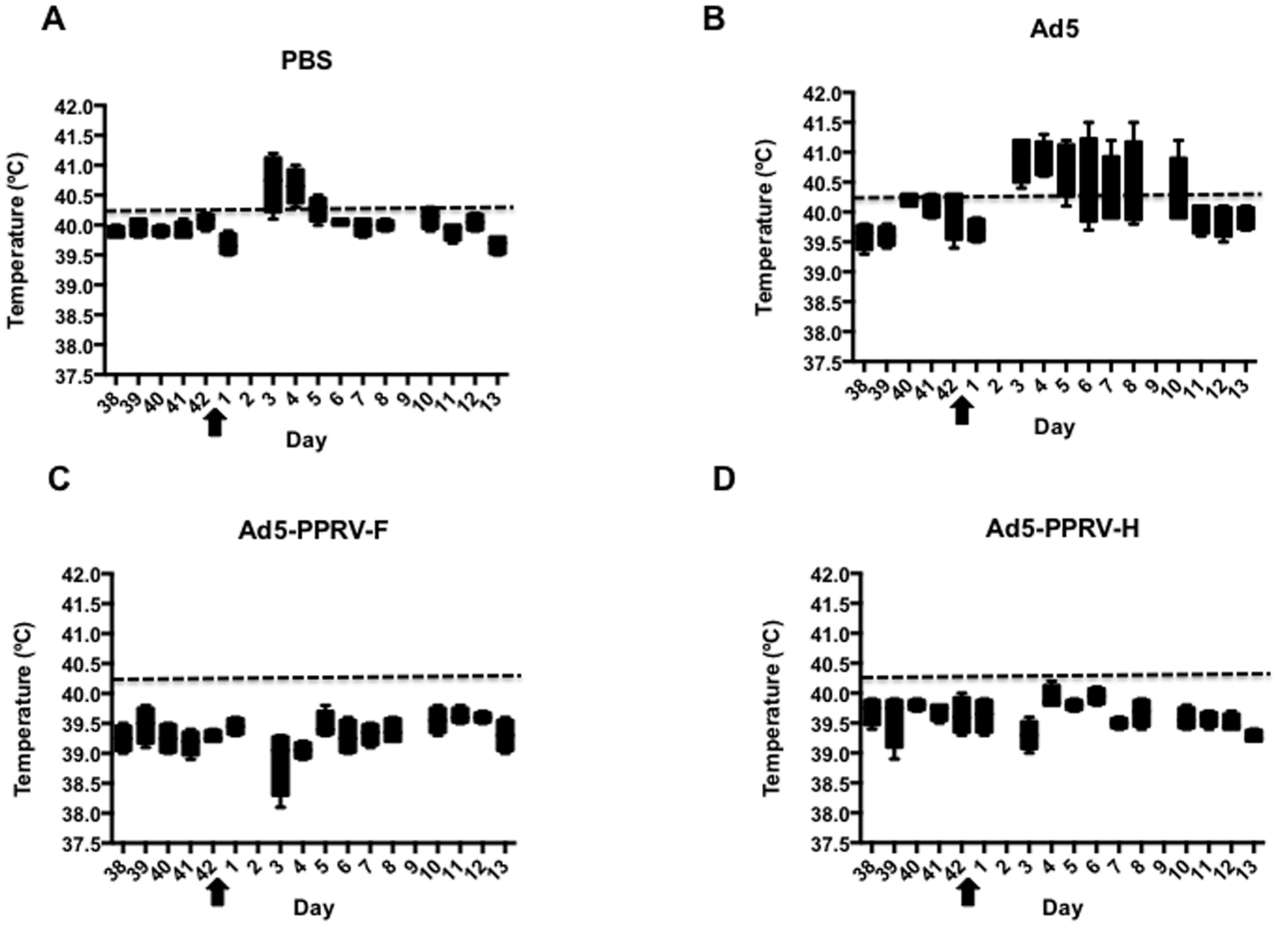
Rectal temperature in vaccinated and non-vaccinated animal groups upon PPRV challenge. The minimum to maximum values of temperatures from day 38 of the experiment to day 13 pc for each experimental group is represented in a box and whiskers plot. Each panel corresponds to each group of animals, as indicated. Arrows indicate the day of PPRV challenge. The dashed line in each panel indicates the threshold temperature value above which animals were considered to have fever.

**Figure 6:**
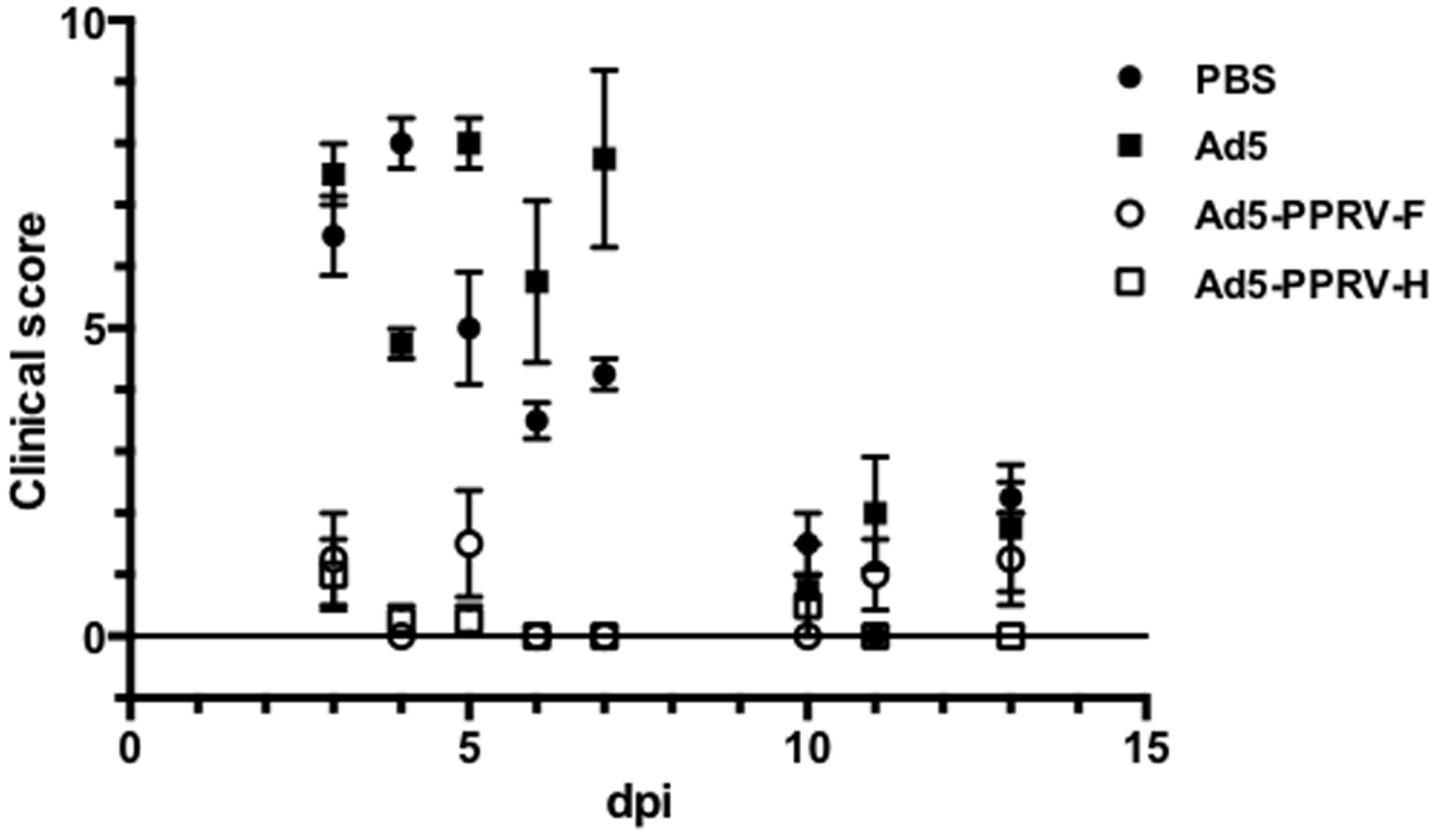
Clinical disease following PPRV challenge. Experimentally inoculated sheep were graded by clinical observation for the development of disease after PPRV challenge with slight variations from the clinical score proposed on [26]. Data are presented as average (+/− SEM) score for different days post challenge (dpi) for each treatment group as indicated in the legend. Differences were found to be significant (p<0.0001; two-way ANOVA) among control (PBS or Ad5-treated) and each vaccinated group from days 3 to 7 post challenge.

To determine the effect of vaccination with either Ad5-PPRV-F or Ad5-PPRV-H in sheep on PPR development, rectal temperature as well as clinical signs were monitored daily after challenge in all experimental groups. Normal rectal temperatures ranged from 38.5–40.3°C and were not affected by adenovirus inoculation (Figure 5). Following inoculation with PPRV, unvaccinated animals consistently developed fever (40.5–41.5°C) after PPRV challenge (Figure 5 A and B), which lasted from days 4 pc to 10 pc depending on the individual. In stark contrast, none of the vaccinated animals developed high temperature (Figure 5 C and D). Clinical signs of disease including mild depression, moderate mucopurulent nasal discharge, red conjunctives, poor appetite, and dull look of wool were detected in unvaccinated animals (groups 1 and 2). By contrast, in vaccinated animals clinical signs were strongly reduced or absent (Figure 6). These data indicate that sheep vaccinated with Ad5-PPRV-F or Ad5-PPRV-H were protected against virulent PPRV challenge.

**Figure 7:**
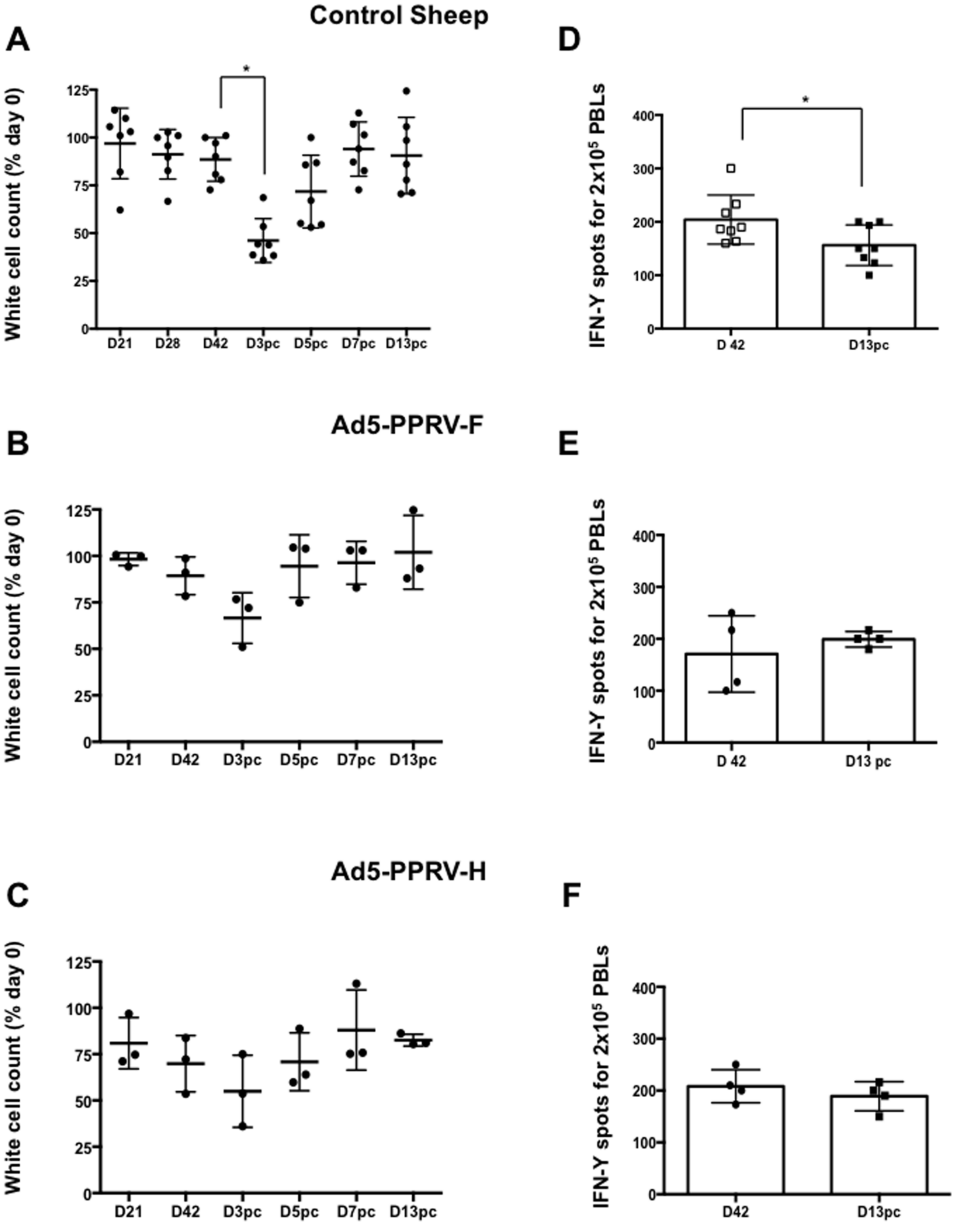
Specific leukopenia and immunosuppression in PPRV infected sheep. Percentage of leukocytes in blood samples obtained at different times from vaccinated (**A**), Ad5-PPRV-F (**B**) or Ad5-PPRV-H (**C**) vaccinated sheep were counted on Auto Hematology Analyzer (Mindray Bc-2800Vet). Each dot corresponds to individual sheep. The asterisks indicate statistically significant (p<0.05, Wilcoxon Test). Production of IFN-γ in response to Con-A stimulation at day 42 post-vaccination and day 13 after challenge in control sheep (groups 1 and 2) (**D**), Ad5-PPRV-F vaccinated sheep (group 3) (**E**), and Ad5-PPRV-H vaccinated sheep (group 4) (**F**). Each dot corresponds to one animal. Bars indicate the average of all animals. A Wilcoxon matched-pairs signed-rank test was used for this analysis (p<0.05).

Infection of sheep with virulent PPRV causes severe leukopenia and lymphopenia during the acute phase of the disease [32]. Therefore, total leukocyte counts were determined in Ad5-PPRV-F or Ad5-PPRV-H vaccinated and unvaccinated animals. At day 3 pc a significant decline in leukocyte numbers was observed in unvaccinated sheep (Figure 7 A), whereas no significant decrease was detected in vaccinated animals (Figure 7 B and C). The other haematological parameters analyzed remained stable throughout the experiment (data not shown). Furthermore, we studied T-cell responsiveness to mitogen stimulation (Con-A) on PPRV challenged animals. In control sheep, which received either PBS or Ad5, the capacity to produce IFN-γ in response to Con-A stimulation was significantly reduced after PPRV infection (p<0.05; Wilcoxon matched-pair signed test) (Figure 7 D). This suggests impairment of T-cell activation during PPRV infection. On the other hand, vaccinated sheep, which had received either Ad5-PPRV-F or Ad5-PPRV-H, did not display significant differences in their response to Con-A after the viral challenge indicating that vaccination was capable of overcoming the immunosuppressive effects of the virus (Figure 7 E and F).

**Figure 8:**
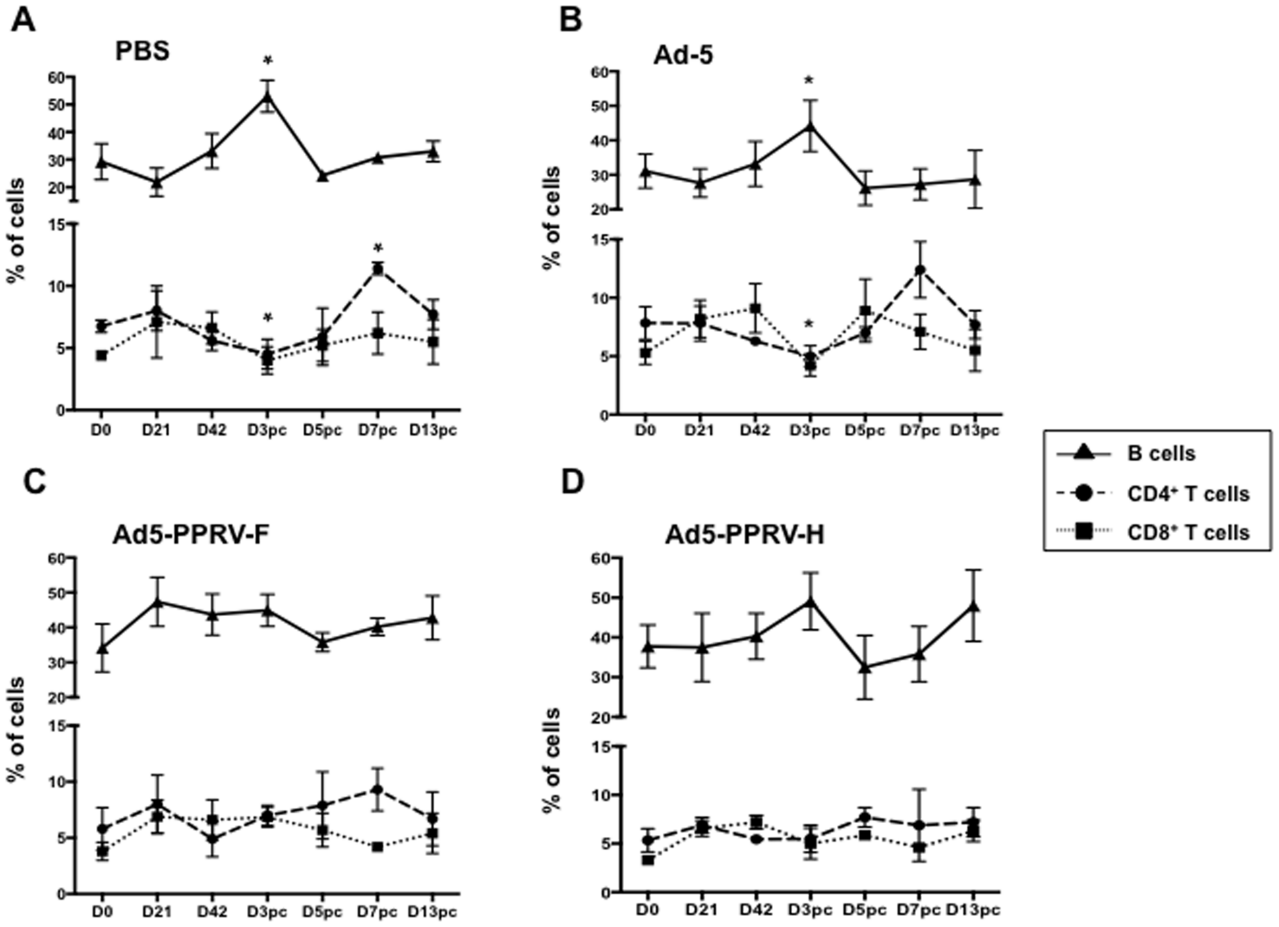
Effect of vaccination with Ad5-PPRV-F and Ad5-PPRV-H on T- and B- cell populations. Lymphocytes were stained with anti-CD4, anti-CD8 and anti-IgM monoclonal antibodies and analyse by flow cytometry. Results are expressed as the percentage of individual animals (each dot corresponds to one animal) and the mean ± SD of B cells (triangle), CD4^+^ T cells (circle) and CD8^+^ T cells (square) lymphocytes from **A**) PBS treated, **B**) Ad-5, **C**) Ad5-PPRV-F and **D**) Ad5-PPRV-H. Asterisks indicate statistically significant by Wilcoxon test (p<0.05).

To determine the lymphocyte population that accounted for the leukopenia in unvaccinated animals, CD4^+^-, CD8^+^-T cells and B-cells were stained in unvaccinated (PBS and Ad-5 inoculated sheep) and Ad5-PPRV-F or Ad5-PPRV-H vaccinated sheep. Unvaccinated animals showed a significant decline in the percentage of both CD4^+^ and CD8^+^ T cells by day 3 pc (Figure 8 A and B). By contrast, neither T cells nor B cells changed their percentage during the experimental time course studied (D0 to D13pc) in vaccinated sheep (Figure 8 C and D).

## Discussion

A need for safe and effective vaccines against PPRV is evidenced by the publication of different strategies using recombinant viral vectors to deliver immunogenic PPRV antigens to goats and sheep. In this regard, adenovirus based vectors show a great potential as they have been reported as successful antigen deliverers [38-39-40] inducing strong mucosal and systemic immunity [33], [34]. Adenoviruses can infect different host species [35], [36] facilitating a long-lived response in animals [37]. The human Ad5 virus is a good candidate to design vaccines for livestock because of the absence of pre-existing immunity against this virus. Moreover, adenoviral vaccines are easy to administer, inexpensive to produce and heat-stable, facilitating convenient means for storage and transport [38], [39], [40].

For their use as PPRV vaccines, different adenoviruses have been proposed. Thus, a replication competent canine adenovirus expressing the PPRV H protein was shown to induce neutralizing antibodies in goats after a single inoculation [15], although the titers obtained were lower than in animals vaccinated with the attenuated conventional vaccine. Replication defective human Ad5 adenoviruses expressing F, H or an F-H fusion protein were also shown to induce both long lasting neutralizing antibodies as well as lymphoproliferative responses in inoculated goats [41]. However, no protection assays were performed in either vaccination study. Similarly, our group reported the potential use as a vaccine for PPRV of the two recombinant replication defective human Ad5 adenoviruses expressing the F or H proteins from PPRV [25], that induced specific humoral and cellular responses to PPRV in mice. In this report, we have extended our previous analyses to sheep, showing that both Ad5-PPRV-F or Ad5-PPRV-H induce humoral and cellular responses in inoculated sheep. Importantly, we show for the first time that both constructs efficiently prevent the replication and spread of a virulent strain of PPRV in sheep. A similarly constructed adenovirus expressing the H protein only has been shown to protect against PPRV infection in goats [42], suggesting that this strategy might be applicable to both species.

Developing effective vaccines to PPRV in sheep is essential as these usually present milder clinical signs of infection than goats [43], which may frequently be overlooked, limiting effective disease control and facilitating PPRV spread. It has been proposed that PPRV replicates primarily in tonsil and lymph nodes rather than in epithelial cells from the respiratory mucosa [26]. Therefore, unlike in previous studies, we chose to inoculate the challenge virus using the intravenous route. As described, this produced mild but consistent signs of disease in non-vaccinated sheep which correlated with the presence of high levels of viral RNA in blood and PPRV presence in swabs, leukopenia and pyrexia, showing that this is a valid model for assessing PPRV vaccines in this species.

In our study, we have shown that delivery of either F or H antigens through the use of recombinant adenoviral vectors is able to elicit protective B and T cell responses. After Ad5-PPRV-F or Ad5-PPRV-H vaccination, anti-PPRV IgG are detected as early as day 7 post-immunization. The IgG levels obtained with the first immunization could be sufficient to protect the sheep, because the titre increase after the booster vaccination on day 21 was only half a logarithm, and these IgG levels were similar to those obtained after PPRV challenge. Neutralizing titers obtained, although slightly lower than 10 before challenge, are sufficient to generate protection, since their presence in sera correlated with the absence of clinical signs observed in vaccinated sheep, probably assisted by the generated T cell response.

While the presence of neutralising antibodies is considered predictive of vaccine efficacy [30], several lines of evidence suggest the importance of an adequate T cell response for prevention of PPRV infection [32], [44]. Here we have shown that specific anti PPRV T cell responses are mounted in all vaccinated sheep by day 42, after the booster inoculation. When T cell responses were assessed on day 21, only one vaccinated sheep displayed specific PPRV proliferative response and no IFN-γ production was detected (data not shown). This suggests that, unlike for the humoral response, booster vaccination with Ad5-PPRV-F or Ad5-PPRV-H is probably needed for the development of a sustained T cell response to the virus. Alternatively, higher doses of inoculated vaccine virus might improve this response. It is important to mention that in our experiments, doses of vaccine virus are on average ten times lower than those used in other adenovirus vaccine studies mentioned above. No significant T cell responses to PPRV in unvaccinated sheep during the mock vaccination period were detected, nor after challenge. Because of the lymphotropism and immunosuppressive effect induced by PPRV [32], T cell responses are likely to be delayed, providing the virus with a window of opportunity where it can replicate and spread. T cell responses are probably mounted in unvaccinated animals after challenge as indicated by the increased production of PPRV-specific IgG, which requires T-cell help for class switching [45]. It is important to note that assessing T cell responses in peripheral blood has limitations in this setting, as PPRV-specific T cells are likely to be recruited at the sites of active infection and in the lymph nodes. Indeed, all unvaccinated sheep were recovering by day 13 pc, suggesting that effective immunity is being generated but because the experiment was stopped at that point, the expansion peak of PPRV-specific T cells detectable in blood was probably missed. The defensive immune response of the sheep against PPRV infection is obscured by the generalized immunosuppression common to the morbillivirus [46], which persist even after the recovery of peripheral white blood cell count. This PPRV immunosuppression in the initial stages of infection, was detected in unvaccinated sheep by a significant decrease in the percentage of white cells counted in blood at day 3 pc compared to day 0 (Figure 7). More precisely the percentage of CD4^+^- and CD8^+^- T cells significantly decreased by day 3 pc coinciding with a significant B cell increase (Figure 8), suggesting that a substantial T-cell-independent B-cell activation response is being promoted in PPRV infected sheep. This phenomenon was not detected in vaccinated sheep. Interestingly, the production of IFN-γ to Con-A was also impaired after challenge in control sheep, whereas no differences were observed in vaccinated sheep. This confirms that T cell responses are likely to be reduced following PPRV infection and that vaccination could overcome this immunosuppressive phenomenon.

Goats inoculated with Ad-F and H vectors developed T cell responses specifically directed towards H antigen derived peptides, suggesting this may be the most important antigen for T-cell mediated protection *in vivo* [42]. However, we have shown that, at least in sheep, expression of F antigen results in specific T -cell responses and that sheep can be protected, too, in the absence of the H antigen. Whether this is a species-dependent phenomenon remains to be established, but it suggests that, for the application as vaccines both antigens should be considered. This is in agreement with the results showing that best immune responses in terms of neutralizing antibodies were achieved with adenovirus expressing an F-H fusion protein in goats [41].

As rinderpest is declared eradicated, a commitment to destroy most remaining stocks of live virus would benefit from the development of safe, alternative rinderpest vaccines [47]. In this sense, it would be important to test whether this or other recently developed PPR vaccines might confer crossprotection.

The results presented here provide an important proof of concept in adenoviral-vectored vaccine for PPRV, demonstrating for the first time in sheep that adenoviruses expressing F or H PPRV proteins generate specific humoral responses with PPRV-neutralizing antibodies as well as T-cell responses against PPRV of different lineages. While further work will determine if this approach proves useful for combating PPRV in the natural hosts on a large scale, we propose these new adenovirus-vectored vaccines as an alternative to conventional vaccines in which the potential risks related to the production of inactivated vaccines and the distinction between vaccinated and infected animals are overcome.
